# Prioritizing Avian Species for Their Risk of Population-Level Consequences from Wind Energy Development

**DOI:** 10.1371/journal.pone.0150813

**Published:** 2016-03-10

**Authors:** Julie A. Beston, Jay E. Diffendorfer, Scott R. Loss, Douglas H. Johnson

**Affiliations:** 1 Geosciences and Environmental Change Science Center, U.S. Geological Survey, Denver, Colorado, United States of America; 2 Department of Natural Resource Ecology and Management, Oklahoma State University, Stillwater, Oklahoma, United States of America; 3 Northern Prairie Wildlife Research Center, U.S. Geological Survey, Saint Paul, Minnesota, United States of America; Institute of Agronomy, University of Lisbon, PORTUGAL

## Abstract

Recent growth in the wind energy industry has increased concerns about its impacts on wildlife populations. Direct impacts of wind energy include bird and bat collisions with turbines whereas indirect impacts include changes in wildlife habitat and behavior. Although many species may withstand these effects, species that are long-lived with low rates of reproduction, have specialized habitat preferences, or are attracted to turbines may be more prone to declines in population abundance. We developed a prioritization system to identify the avian species most likely to experience population declines from wind facilities based on their current conservation status and their expected risk from turbines. We developed 3 metrics of turbine risk that incorporate data on collision fatalities at wind facilities, population size, life history, species’ distributions relative to turbine locations, number of suitable habitat types, and species’ conservation status. We calculated at least 1 measure of turbine risk for 428 avian species that breed in the United States. We then simulated 100,000 random sets of cutoff criteria (i.e., the metric values used to assign species to different priority categories) for each turbine risk metric and for conservation status. For each set of criteria, we assigned each species a priority score and calculated the average priority score across all sets of criteria. Our prioritization system highlights both species that could potentially experience population decline caused by wind energy and species at low risk of population decline. For instance, several birds of prey, such as the long-eared owl, ferruginous hawk, Swainson’s hawk, and golden eagle, were at relatively high risk of population decline across a wide variety of cutoff values, whereas many passerines were at relatively low risk of decline. This prioritization system is a first step that will help researchers, conservationists, managers, and industry target future study and management activity.

## Introduction

In recent decades, concerns about climate change and energy independence have driven rapid growth in renewable energy production in the United States (US). In 2012, wind energy was the leading source of new electricity generation capacity in the US [1]. Although wind power avoids many of the adverse environmental effects associated with fossil fuel use, it is not completely impact-free. In particular, wind turbines displace and kill a variety of wildlife, which has made wind energy a major conservation and policy concern worldwide [2].

Whether or not wind energy has population-level consequences for wildlife species is a critical issue when developing strategies for avoiding, minimizing, and mitigating impacts of energy production. In both the US and the European Union, legal directives focus on the protection of populations. For example, the Eagle Conservation Plan Guidance document developed by the US Fish and Wildlife Service is designed to issue “take” permits for eagles so that fatalities from wind energy are “consistent with the goal of a stable or increasing breeding population” (see page iii in[3]). Similarly, in the European Union, the Birds and Habitat Directive has an overall objective to maintain and restore populations of birds, and wind facilities are regulated in accordance with these standards [4,5]. Scientists have attempted to understand population-level effects of wind facilities through field studies of species demography [6,7], demographic modeling [8–10], and the estimation of mortality thresholds [11]. In addition, numerous studies have quantified turbine collision rates [12–14], investigated bird movements near proposed and existing turbines [15–17], and assessed geographic overlap between species distributions and turbines [18–22]. Nonetheless, to date, no systematic, large-scale, and multi-species assessment frameworks have been developed that combine this information to evaluate the likelihood of population declines from wind energy and to prioritize the bird species at greatest risk.

Prioritization of species with respect to current population status, trend, and threats is commonly used in conservation practice [23,24], and several recent studies have compared impacts of collisions with wind turbines and other anthropogenic structures across avian species. Desholm [25] presented a prioritization system for birds at proposed individual wind facilities that incorporated field surveys of bird use and life history information. Others have compared species’ mortality rates at wind facilities and communication towers by dividing estimates of annual fatalities by continent-wide population estimates [26,27]. These comparisons are not as useful across diverse taxonomic groups that may respond differently to mortality. Another approach used the relationship between the number of collisions and total population size to identify “super colliders” and “super avoiders,” species at much greater or lesser risk of collisions than average [28], but as with the previous approach, the inability to put the collisions in the context of risk of population consequences remains a drawback. Furthermore, all of these approaches focus only on collision fatalities and do not capture potential population declines from indirect effects of changes in habitat or behavior.

We present a method to prioritize bird species based on their risk of population-level impacts from wind energy development. Our method takes into account the current conservation status of a species and the risk it faces due to wind facilities. We considered both direct effects of turbine-caused fatality and indirect effects of habitat modification. Moreover, we included natural history information to account for expected variation in effect sizes across species with different life histories and levels of habitat specialization. We applied our method to avian species breeding in the US, but with appropriate parameter estimates, this framework is applicable to birds in other geographic regions impacted by wind energy development.

## Methods

### Overview of prioritization approach

The prioritization approach is designed to be used for avian populations at a regional to national scale. Because we relied on available data, we restricted our analysis to those species of birds that use the US for breeding and are detected in the North American Breeding Bird Survey (BBS; www.pwrc.usgs.gov/bbs/). Therefore, the final prioritization score applies to the US breeding population, and it only estimates the potential effects of turbines in the US breeding range, not all turbines in the species’ entire range.

**Fig 1:**
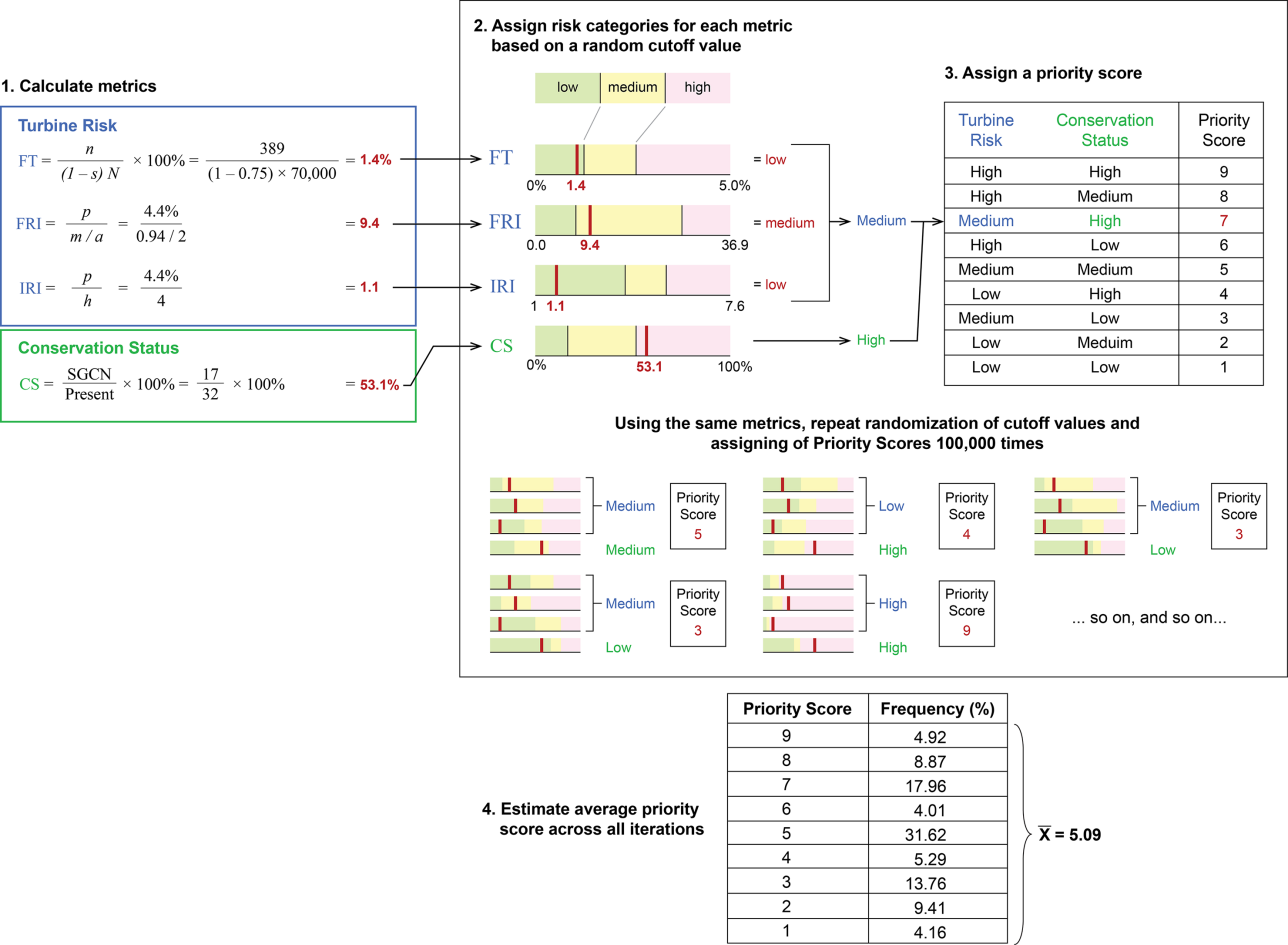
Prioritization work-flow. Prioritization for each species (in this example, the ferruginous hawk) begins with the calculation of 3 turbine risk metrics and 1 conservation status metric. Then random cutoff values are repeatedly drawn to assign each metric value to a low, medium, or high category. These categories are used to determine a priority score for each iteration, which can be summarized across all iterations.

The prioritization approach included 4 main steps. First, we calculated values for four metrics for each species—one metric for conservation status and three for turbine risk (Fig 1, Step 1, metrics described below). We then used a Monte Carlo approach to estimate a “priority score” for each species (Fig 1, Steps 2 and 3) and developed a species prioritization list according to this average score (Fig 1, Step 4). A species’ current conservation status should indicate how capable it is of withstanding additional mortality or habitat loss. The three metrics for ‘turbine risk’ quantify a species’ susceptibility to effects from wind energy. Species are categorized as high, medium, or low for both conservation status and turbine risk, and then these categorizations are combined (Fig 1, Step 3). The combinations result in risk scores from 1 to 9 for each species (with 1 corresponding to lowest risk and 9 corresponding to highest risk). The scoring system weighs turbine risk over conservation status. For example, a species with high turbine risk and medium conservation status is scored an 8, while a species with medium turbine risk and high conservation status is scored a 7. Using a combination of current conservation status and turbine risk allows accommodation of species that are already imperiled, perhaps due to other factors, and addressing cumulative risk from turbines and other threats. This approach follows previous prioritization methods that combine conservation status with an assessment of the factor(s) causing additional risk [23,29].

### Current Conservation Status

We expect species with an elevated conservation status to experience greater cumulative effects of (but not necessarily exposure to) wind energy development because they often have a small population size [30,31], restricted range [32], ongoing population decline or habitat loss [31], or face current or future anthropogenic threats such as harvest, climate change, and land use change [33,34]. Because we sought to use a numerical measure of conservation status, we used the percentage of states in which a species occurred where it was considered a Species of Greatest Conservation Need (SGCN) in a State Wildlife Action Plan [35]. This measure of conservation status does not account for federal listing under the Endangered Species Act or on the US Fish and Wildlife Service list of Birds of Conservation Concern; nonetheless species that were federally listed were also listed as SGCN in an average of 44% of states in which they occurred, compared to just 18% for all other birds. Therefore, in general, federally listed species had higher than average state-level conservation status. Furthermore, federally listed birds will continue to receive attention and protection regardless of the prioritization rank assigned using our methods; therefore, this prioritization approach will augment federal lists with species that may be at risk but not yet listed.

### Turbine Risk Assessment

Many approaches for categorizing risk to species or ecosystems follow risk assessment procedures that incorporate two key measures: (1) the probability of impact, usually described by the area or proportion of individuals affected by a threat, and (2) the magnitude of impact [23,36–38]. We used 3 different approaches to categorize risk associated with wind turbines using this two-pronged view of risk (Table 1).

**Table 1:** The risk metrics used in the prioritization, their equations, and data required.

Risk Metrics	Equation	Data required
Proportion of fatalities due to turbines (FT)	$\text{FT}=\frac{n}{(1-s)\;N}$	*n*, the number of individuals killed by turbines annually *s*, adult survival rate *N*, total population size
Fatality Risk Index (FRI)	FRI=pma	*p*, percent of a species population living near wind turbines*m*, maternity, female offspring per female per year *a*, age at first reproduction
Indirect Risk Index	$\text{IRI}=\frac{p}{h}$	*p*, see above *h*, the number of habitats a species uses

### Proportion Fatalities Due to Turbines

The most obvious and well-studied effect of wind energy generation on wildlife is fatalities of birds and bats from collisions with turbines. Various studies have quantified fatalities at individual wind facilities [39,40], and researchers have projected fatality rates to larger scales [14,41]. Many wind installations cause few fatalities, and while species with high inherent rates of population increase may be able to withstand these losses, the extra fatality could be significant for species with slow life histories [8,42]. In general, long-lived species with low mortality rates are more likely to experience additive mortality from anthropogenic sources than short-lived species with naturally high mortality [43]. Therefore, we reasoned that species with a high proportion of their total annual fatalities due to wind facilities (i.e., animals killed at wind facilities/animals that die from any cause) are at higher risk of population consequences from wind energy. The increased risk could occur either because the number of fatalities caused by wind turbines (the numerator) is excessively high or because the total number of annual fatalities (the denominator) is low and the population is sensitive to changes in mortality.

To estimate species-specific turbine fatalities, we multiplied the estimate of total avian fatalities in the conterminous US from wind turbines by the proportion of observed fatalities attributed to each species adjusted for differences in detection rate among size classes. This approach has been used previously without the size adjustment for passerines in North America [26] and birds in Canada [44]. The fatality data were taken from a meta-analysis of avian wind collision fatalities at 53 wind facilities across the US [14], and we divided the number of observed carcasses by the searcher detection rate for each species’ size class [45]. Our approach assumes that the sample of wind facilities with fatality data is a random geographic sample and that body size explains any differences in detectability among species. While these assumptions are violated by the existing data, no other information is currently available to assess species-specific fatality at a national scale. We gathered estimates of population size within the US from Partners in Flight, the Mid-Winter Waterfowl Survey, and Waterbird Conservation for the Americas. We used Birds of North America [46], the Institute for Bird Populations [47], and review papers [48,49] to collect estimates of adult survival rate for each species. To calculate the proportion of fatalities due to turbines (FT), we divided the estimate of the number of individuals killed by turbines annually (*n*) by the product of population size (*N*) and the adult mortality rate (calculated as 1 –adult survival rate, *s*):

$$\text{FT}=\frac{n}{(1-s)\;N}$$

This metric combines the proportion of the population affected with the severity of the effect, producing a value that is comparable across species.

### Fatality Risk Index

We developed the Fatality Risk Index (FRI) to estimate the difference in risk from wind between long-lived, slow reproducing species and short-lived, rapidly reproducing species. This index may yield useful information about relative risk when actual estimates of annual fatalities from wind turbines or other sources are unavailable. The FRI is the percent of a species’ population living in the vicinity of wind turbines (*p*) divided by an index of life history speed. To assess the probability of impact, we estimated the proportion of a species’ US breeding population that occupies areas that include wind turbines using the BBS range maps (U.S. Geological Survey Patuxent Wildlife Research Center; available at www.mbr-pwrc.usgs.gov/bbs/). These maps are constructed with a grid of 21.475 km by 21.475 km cells, with a relative abundance value in each cell. For each species, we multiplied the abundance index value assigned to each cell in the US portion of the BBS range map by the area of that cell (461 km^2^, except for border and coastal cells). We then standardized this value to sum to 1 across all cells in the US to calculate the percent of the US population that occurs in each cell. We summed the resulting population percentages across cells in the US that contained at least one wind turbine. A significant drawback to this approach is the exclusion of migratory pathways or wintering habitat that may contain turbines. Though we would prefer a better estimate of the exposure of bird species to wind facilities throughout their annual cycle, detailed maps of distribution and use throughout the year that are comparable across many hundreds of species are not available. Nonetheless, our approach is adaptable to inclusion of this type of information as it becomes available.

To account for the differences in response to fatalities for demographically “fast vs. slow” species, we modeled the tempo of a species’ life history using the ratio of maternity (female offspring per adult female per year, *m*) to the age at first reproduction (*a*) [50,51]. For birds and mammals, *m*/*a* is correlated not only with many demographic parameters (e.g., juvenile and adult survival, fecundity), but also the elasticity of population growth rate to these parameters [50,51]. Moreover, these values are relatively easy to find in the literature for most species. We used the information in the Birds of North America [46] to estimate *m* and *a*. We calculated *m* as the product of clutches per year, nest success, clutch size, hatchability, and a presumed proportion of female offspring of 0.5. When data were lacking for a species, we assumed hatchability was the mean from other species (0.9, 92% of species) and the number of clutches per year was the mode from other species (1, 2% of species). Many altricial species are presumed to begin reproduction during the first breeding season after they fledge. We incorporated this presumption in the absence of direct evidence. We then divided the percent of the population exposed (*p*) by this measure of life history speed to calculate a Fatality Risk Index (FRI) for each species.

FRI=pma

Higher values of this index correspond to higher risk either because a large percentage of the population is exposed or because the life history is slow.

### Indirect Risk Index

Bird species that occur in and around wind facilities may infrequently collide with turbines [52] yet still experience population consequences due to disturbance, displacement, and habitat fragmentation and loss [16,53–55]. For such species, information about their natural history may help categorize risk. A number of studies indicate highly specialized species, those with more restricted geographic ranges, using fewer habitats, or having more restricted diets, are more sensitive to changes in habitat than generalist species [56–62].

To quantify indirect risk, we treated the number of habitats a species uses as an index of niche breadth for each species. Ideally, measures of niche should include some information on the relative use and availability of resources, but these data are unavailable for most species and areas. We used the IUCN habitat categorization information to delineate 30 habitat types (S1 Table), and we recorded the number of habitats considered suitable for each species from the IUCN’s classification database [33]. We divided the same measure of population exposed to wind facilities, *p*, by the number of suitable habitats, *h*, to produce an Indirect Risk Index (IRI).

$$\text{IRI}=\frac{p}{h}$$

Higher values of this index correspond to higher risk because a larger proportion of the population is exposed or because the species has restricted habitat requirements.

### Overall Prioritization

Assigning species to prioritization categories requires the use of cutoff criteria for each metric’s high, medium, and low risk categories. Although designation of cutoffs is common practice in prioritization approaches, these cutoffs are somewhat arbitrarily selected and often blend scientific information with policy and risk aversion considerations. For example, the IUCN criteria for categorizing species into levels of endangerment went through six revisions from 1991 to 2001 [33].

To avoid choosing particular, arbitrary criteria for the high, medium, and low risk categories, we generated 100,000 sets of random cutoff values and used these in the prioritization process (Fig 1, Step 2). The Monte Carlo simulation was done using the runif command in R [63]. Pairs of cutoff values were randomly sampled from a uniform distribution bounded by the minimum and maximum observed values for each metric. Thus, cutoffs were generated separately for conservation status and each of the three turbine risk metrics at each iteration. For all four metrics, the lower value in the pair of randomly selected cutoff values was used to separate low and medium risk categories, and the higher value separated medium and high risk categories.

For each iteration of the Monte Carlo selection, we compared the estimated metric values for each species to the randomly generated cutoff values to assign a high, medium, or low risk category for each metric (Fig 1, Step 2). For turbine risk, a species could be evaluated using any combination of the three turbine risk metrics, depending on the data available. If more than one of the turbine risk metrics were calculated, we used the highest risk category (Fig 1, Step 2). For example, if a species were categorized as high for FT, but medium for IRI, its turbine risk was considered high. Once the species had been given a turbine risk category and conservation status category, we assigned it a risk score from one to nine following Table 2 (Fig 1, Step 3). For each species, we then calculated (across all 100,000 iterations) the percentage of times the species was assigned to each score, the most frequently assigned score, and the average risk score (Fig 1, Step 4). We generated our final list of species rankings based on this average risk score.

**Table 2:** Overall priority assignment.

Turbine risk	Conservation status	Risk Score
High	High	9
High	Medium	8
Medium	High	7
High	Low	6
Medium	Medium	5
Low	High	4
Medium	Low	3
Low	Medium	2
Low	Low	1

Numerical priority rank assigned to avian species based on their relative risk from wind turbines and their current conservation status. Note that this ranking system places higher weight on turbine risk than on conservation status.

Finally, we summarized results by taxonomic orders. This step facilitated comparison with studies from other regions and with species we did not assess, and it also allowed us to assess whether groups that are typically considered high risk (e.g., raptors) actually scored higher than other groups. Furthermore, these summaries give an overview of the output without delving into species-specific detail.

Conservation status was readily available for all species, but a lack of data prevented us from calculating all three turbine risk metrics for every species. Instead, we calculated FT for 166 species, FRI for 179 species, and IRI for 418 species. Because a species could be evaluated with any combination of the three turbine risk metrics, including only a single metric, we were able to assign priority scores to 428 avian species that occur in the lower 48 states (S2 Table). We first present results for individual turbine risk metrics, then the overall prioritization scores.

## Results

### Turbine Risk Metrics

**Fig 2:**
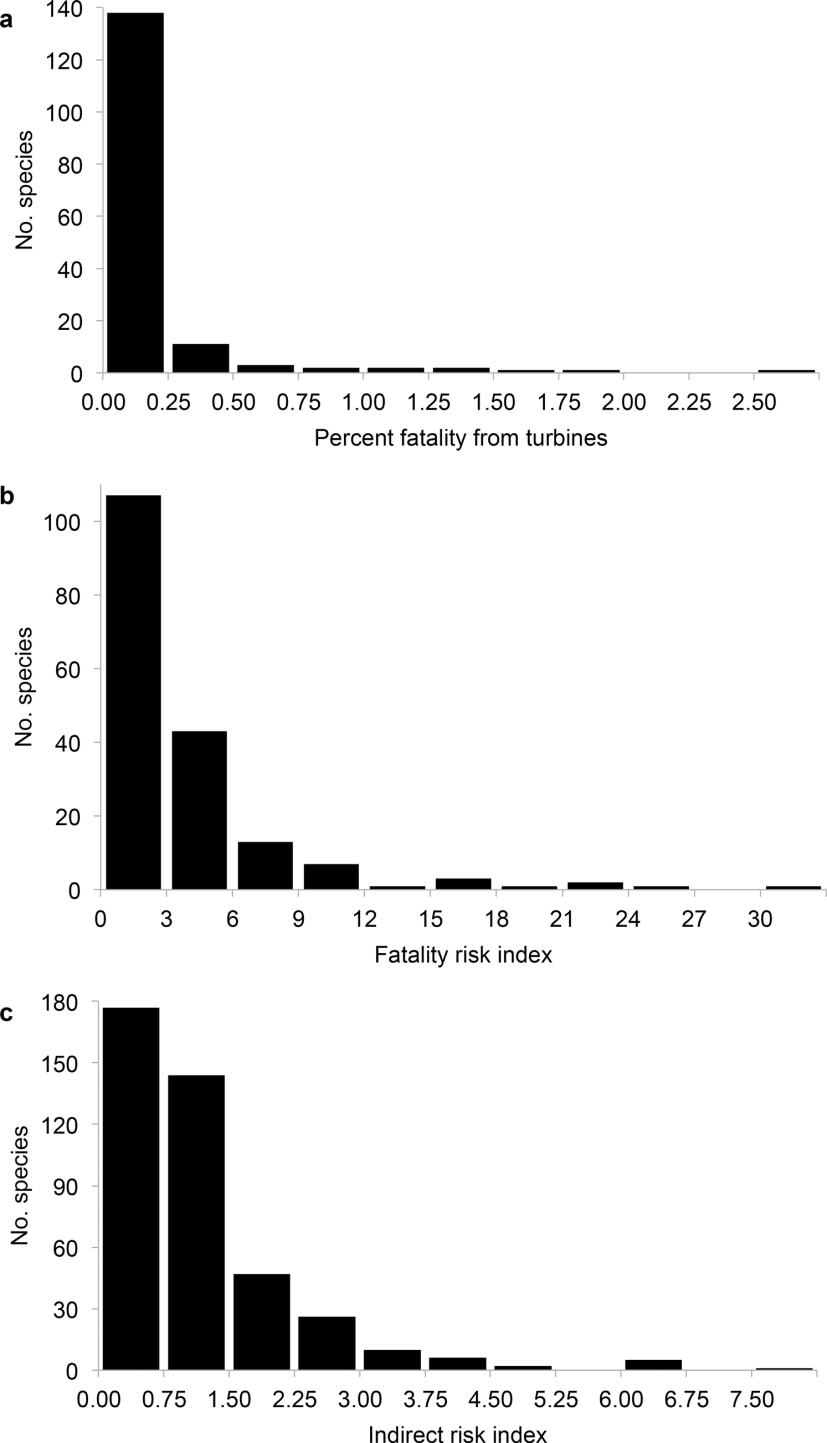
Turbine risk metric distributions. Frequencies of the (a) percentage of total US fatality due to wind turbines for 166 North American bird species, (b) Fatality Risk Index for 179 North American bird species, and (c) Indirect Risk Index for 418 North American bird species.

The distributions of all three turbine risk metrics were right-skewed, with many more species at the low end of the risk spectrum than at the high end (Fig 2). The percentage of all US fatalities accounted for by turbines (FT) was <0.05% for most species (Fig 2A). Species with >1.5% of annual US fatalities attributable to turbines included the long-eared owl (*Asio otus*), golden eagle (*Aquila chrysaetos*), and gray partridge (*Perdix perdix*) (S2 Table, Column J). For FRI, species with high values included the great black-backed gull (*Larus marinus*), Swainson’s hawk (*Buteo swainsoni*), great blue heron (*Ardea herodias*), and Harris’s hawk (*Parabuteo unicinctus*) (S2 Table, column K). The blackpoll warbler (*Setophaga striata*) also had a high FRI value despite a relatively high ratio of maternity to age at first reproduction. This warbler breeds primarily in Canada but has a small breeding population in the northeastern US that overlaps several wind facilities, resulting in >30% of its US breeding population exposed to turbines. The high overlap with turbines also resulted in the blackpoll warbler having the highest IRI value of any species analyzed. Other species with high IRI values included the long-billed thrasher (*Toxostoma longirostre*), sora (*Porzana carolina*), pyrrhuloxia (*Cardinalis sinuatus*) and California thrasher (*Toxostoma redivivum*).

**Fig 3:**
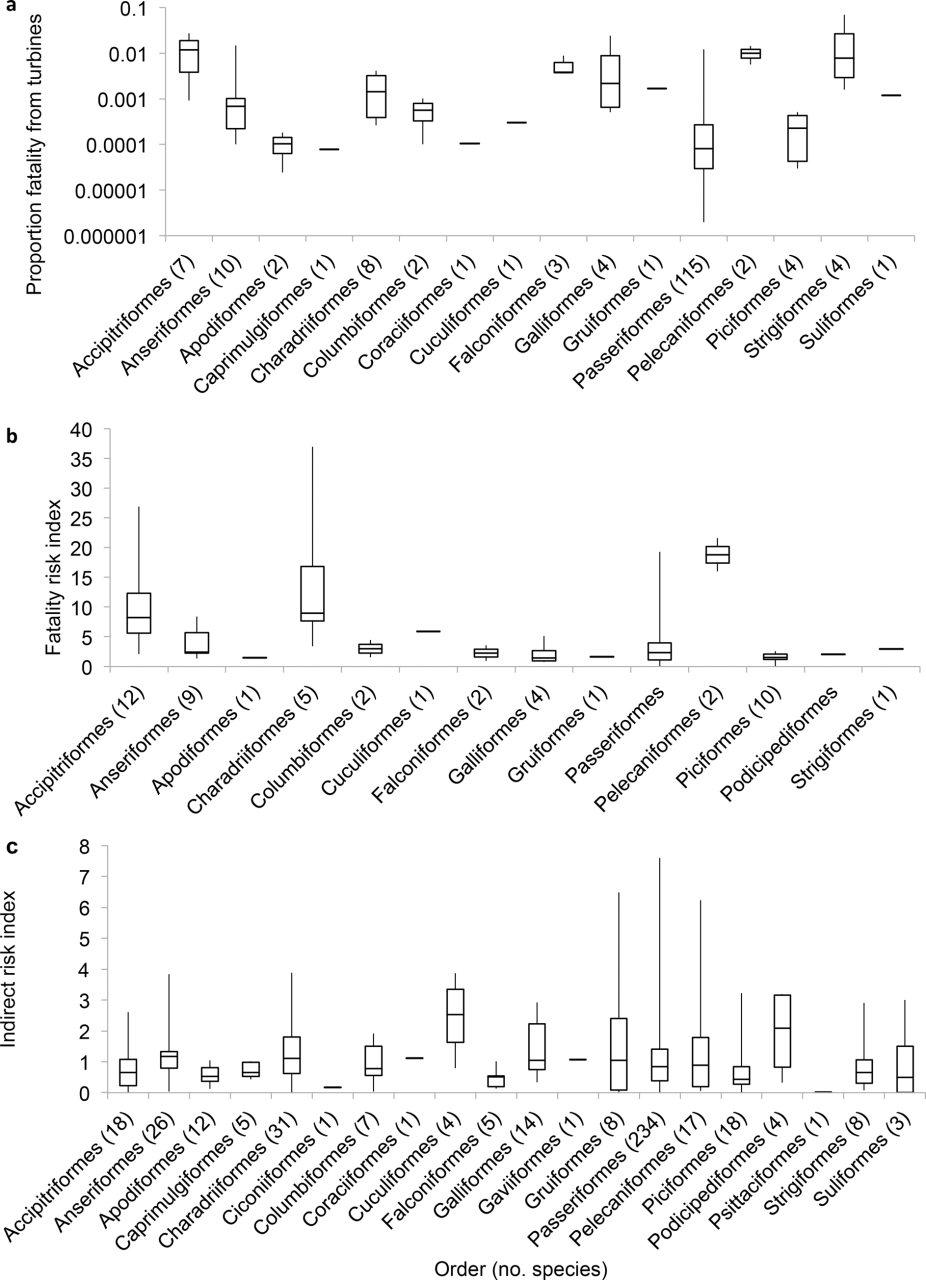
Turbine risk metrics by order. Box plots showing the distribution of (a) proportion of fatality caused by turbines on a log scale, (b) Fatality Risk Index, and (c) Indirect Risk Index within avian orders. Whiskers span the minimum and maximum observed values, the box encloses the middle 50%, and the horizontal line indicates the median value.

The values for the turbine risk measures varied considerably among, and within some, taxonomic orders (Fig 3). Accipitriformes and Pelecaniformes had two of the three highest means for both fatality metrics, with Strigiformes and Charadriiformes in the top three for FT and FRI, respectively. Cuculiformes and Podicipediformes had the highest order means for IRI, but Gruiformes, Passeriformes, and Pelecaniformes had individual species with higher IRI values.

### Overall Prioritization

**Fig 4:**
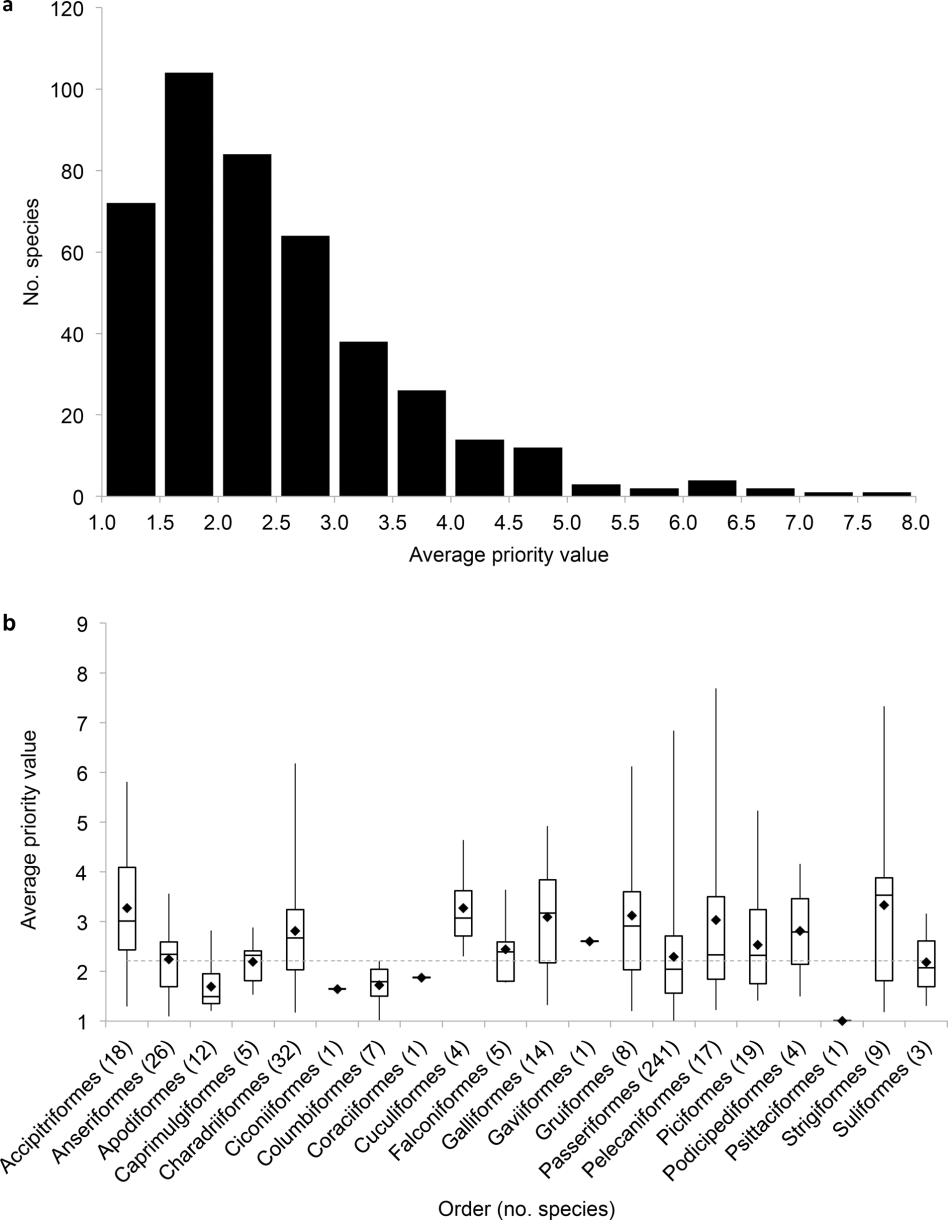
Overall priority results. The distribution of average overall priority score (a) across avian species and (b) within avian orders for 428 species that occur in the conterminous US. Higher values indicate higher priority and greater potential risk from wind facilities.

In the overall prioritization, many species were consistently ranked low priority regardless of the cut-off criteria (Fig 4A). Only 40 species had an average priority score of at least four (Fig 4A, Table 3), while 325 species had an average priority score less than three (Fig 4A). The species with the highest average priority scores were not a representative sample across orders (Fig 4B). In particular, Accipitriformes had a higher median priority than all species combined (Fig 4B), and 39% of Accipitriformes had average priority scores of four or above. On the other hand, passerines had a low median priority (Fig 4B), and only 7% of species in this order had average priority scores of four or above.

**Table 3:** High priority avian species.

		Average	Percent of criteria sets in which elevated turbine risk was due to:			
Common name	Order	Priority (SD)	FT		FRI	IRI
American Bittern	Pelecaniformes	7.69 (1.60)				97
Long-eared Owl	Strigiformes	7.34 (1.14)	100			
California Thrasher	Passeriformes	6.84 (1.87)				97
Long-billed Thrasher	Passeriformes	6.51 (1.78)				98
Blackpoll Warbler	Passeriformes	6.32 (0.75)	0		27	100
Great Black-backed Gull	Charadriiformes	6.18 (0.58)	0		100	17
Sora	Gruiformes	6.12 (1.82)				98
American Woodcock	Charadriiformes	6.07 (2.10)	1		28	74
Swainson's Hawk	Accipitriformes	5.81 (2.03)	13		91	13
Black-crowned Night-Heron	Pelecaniformes	5.67 (2.12)	11		64	36
Pyrrhuloxia	Passeriformes	5.40 (1.66)				97
Great Blue Heron	Pelecaniformes	5.33 (2.11)	25		80	18
Golden-fronted Woodpecker	Piciformes	5.23 (1.62)				38
Greater Sage-Grouse	Galliformes	4.92 (1.47)				29
Herring Gull	Charadriiformes	4.91 (1.43)	15			16
Le Conte's Thrasher	Passeriformes	4.89 (2.15)				53
Scaled Quail	Galliformes	4.88 (2.19)				56
Ferruginous Hawk	Accipitriformes	4.87 (2.12)	41		41	24
Golden Eagle	Accipitriformes	4.72 (2.12)	43		64	6
Black-chinned Sparrow	Passeriformes	4.68 (2.21)				84
Grasshopper Sparrow	Passeriformes	4.67 (1.96)	0	21		31
Black-billed Cuckoo	Cuculiformes	4.64 (2.21)		25		65
Harris's Hawk	Accipitriformes	4.61 (1.95)		76		41
Cerulean Warbler	Passeriformes	4.56 (2.01)	2	22		30
Nelson's Sparrow	Passeriformes	4.50 (2.15)		47		52
Virginia Rail	Gruiformes	4.45 (2.26)				77
Henslow's Sparrow	Passeriformes	4.31 (1.95)				27
McCown's Longspur	Passeriformes	4.29 (2.27)				71
Greater Prairie-Chicken	Galliformes	4.27 (2.21)				54
Upland Sandpiper	Charadriiformes	4.23 (2.06)				42
Bobolink	Passeriformes	4.20 (2.13)	4	25		43
Pied-billed Grebe	Podicipediformes	4.16 (2.21)		9		66
Bronzed Cowbird	Passeriformes	4.16 (1.79)				89
Northern Pygmy-Owl	Strigiformes	4.15 (0.65)				5
Sedge Wren	Passeriformes	4.14 (2.11)		12		43
Cactus Wren	Passeriformes	4.14 (2.22)		4		68
Bald Eagle	Accipitriformes	4.11 (0.84)				6
Seaside Sparrow	Passeriformes	4.07 (1.78)		5		24
Northern Harrier	Accipitriformes	4.04 (1.95)	10	23		14
Osprey	Accipitriformes	4.00 (2.07)	31	29		5

Avian species with average overall priority ≥4 based on 100,000 sets of random criteria.

## Discussion

Our prioritization approach incorporates multiple sources of information and different types of risk to assess the potential population-level consequences of wind energy development on birds in the conterminous United States. The use of sets of random cutoff criteria for category assignments avoids the need for arbitrary criteria-setting decisions and gives insight into the level of uncertainty associated with a species’ status. The vast majority of species were consistently ranked as having low priority, regardless of the selected cutoff values, and thus our approach allows researchers, managers, and industry to narrow their focus to those species most likely to experience population declines due to wind facilities.

Species assigned to high priority categories were not representative of all birds considered, but generally reflected differences in susceptibility stemming from the natural histories of different taxa. For instance, birds of prey generally have low natural mortality rates and smaller population sizes due to their position as apex predators. Birds of prey that we identified to be at greatest relative risk of experiencing population declines from wind energy included the long-eared owl, Swainson’s hawk, ferruginous hawk, and golden eagle. Notably, the golden eagle has been the focus of much concern and debate about wind energy development due to its suspected susceptibility to population decline. Charadriiformes and Pelecaniformes are two orders that also include long-lived species that ranked highly, many of which are also habitat specialists. While passerines typically have fast life histories, a few species, like the California thrasher and long-billed thrasher, ranked high due to the large proportion of their range exposed to wind turbines and their specialization on one or two habitat types.

The use of multiple metrics in the turbine risk element of our prioritization causes species to be highly ranked for different reasons. This was a conscious decision to combine conservation status with both direct (FT and FRI metrics) and indirect (IRI metric) effects of wind energy into a single prioritization rank. For example, the American bittern, the highest ranked species, was assessed using only the IRI. This species ranked high because the relatively high (80%) percent of states considering it a species of greatest conservation need resulted in many iterations with a high value for conservation status. In addition, the American bittern uses only 1 habitat in the adapted IUCN classification; so despite a moderate level of overlap with turbines (6.22%), its IRI value was relatively high. In contrast, the long-eared owl ranked high because the percentage of annual fatality caused by turbines (FT) was calculated at ~5%. Both of these species deserve additional attention, but for very different reasons. The American bittern may be impacted by wind energy because it is a habitat specialist and some of its habitat overlaps with wind turbines. For long-eared owls, collisions with wind turbines caused the highest percentage of annual fatality for any bird species assessed.

The collection of more detailed data on highly prioritized species, either through a literature search or field work, would be a logical next step for assessing wind energy impacts. In our prioritization approach, the reason a species ranks highly should determine the types of information required to more fully evaluate its risk from wind energy. In general, species ranked highly for IRI should be evaluated to discover if, and by how much, current facilities are displacing individuals and the mechanism of this displacement (e.g., behavioral avoidance, habitat loss). By examining studies on bird avoidance of operating facilities, it may be possible to filter out species with a high Indirect Risk Index that actually tolerate wind turbines well.

Although species ranked highly via FRI are at potential risk given their overlap with turbines and their life history speed, some may avoid turbines or simply not interact with them. High FRI species need to be evaluated for their risk of collision. For example, the American crow (*Corvus brachyrhynchos*) is a large, widely distributed bird that is unlikely to be overlooked during fatality monitoring, and it has a low FT value because few individuals have been observed dead at turbines. It does, however, co-occur with turbines and have a relatively slow life history, giving it a high FRI value. The monitoring evidence suggests that this high value probably incorrectly categorizes fatality risk. Species like the American crow, with a high FRI and low FT, may be successfully avoiding collisions. Likewise, studies that identify species attracted to turbines could indicate higher risk for species previously considered low priority.

Finally, species with a high FT are at risk because collisions with turbines are accounting for a relatively high percentage of the species annual fatality. In these cases, demographic modeling or modeling of sustainable take limits [64] may be required to determine if the fatalities caused by turbines will impact a species’ status or trend. Thus, further study of prioritized species is necessary before conclusions about actual risk from wind facilities can be drawn. For species that are clearly classified as a high priority, follow-up data collection and population modeling can be done to provide a more refined picture of the magnitude of wind energy’s population-level effects.

Although data on population level effects of wind facilities on wildlife are scarce, available evidence suggests the turbine risk metrics did identify species likely to be affected. For example, many Accipitriformes scored highly on one or both fatality risk metrics, and studies on several Accipitriformes in Europe have found reduced abundance or abandonment of territories near wind facilities [6,65,66]. A meta-analysis of changes in bird abundance before and after turbine installation found Eurasian Charadriiformes showed significant declines in abundance [67], and the North American members of this order had generally high values of FRI. The same study found little evidence of impact on passerines as a whole [67], and the average turbine risk values for passerines were low. Despite the lack of sufficient direct evidence to determine what values of the turbine risk metrics correspond to elevated actual risk, the use of sets of random criteria ensured that relative rankings were robust to categorization uncertainty.

While some of the identified high priority species are not surprising based on concerns that have been previously raised by researchers and conservation groups, our approach also highlights several previously unconsidered species that are potentially at risk from wind energy development. For instance, we found that 18 species were ranked as a higher priority than golden eagles based on their likelihood of experiencing population declines. Although fatalities of golden eagles have resulted in fines for some wind energy companies [68], required monitoring programs at some facilities [69], and spurred large amounts of research on interactions between golden eagles and wind facilities [65,70–72], many of these other highly ranked species have had comparatively little attention. Given a lack of understanding of the interactions between wind energy and birds and limited knowledge of the population status and trends for most bird species, we suggest that our relatively simple and objective approach can aid in generating a first-pass species prioritization result. Future iterations and refinements of our approach can be made as new information about collision fatalities, indirect effects on bird habitats, and bird population status become available.

There are several limitations to the prioritization approach, and we expect further refinements to improve turbine risk assessments. One limitation is data on species’ distributions. We relied on distributional data from the Breeding Bird Survey to measure the proportion of the population exposed. These data are coarse in scale and are not well-suited for many nocturnal birds, arctic nesters, and Hawaiian or Alaskan birds. Furthermore, assuming a single turbine in a grid cell has the same potential impact as 100 turbines in a cell is obviously unrealistic. Perhaps even more problematically, the range maps we used exclude migratory pathways and overwintering locations where many species encounter turbines. The measure of the proportion of the population exposed to turbines could be improved through more detailed modeling of species’ distributions through time (e.g., using dynamic distribution maps produced with eBird data, or—for target species—migratory connectivity data obtained from extrinsic or intrinsic markers [73]).

Other data limitations may also affect the output of our prioritization approach. In particular, FT would be better estimated with species-specific fatality data. Though we corrected for differences in detectability among body sizes, estimates derived from species composition of observed fatalities may still be biased by geographic coverage of fatality studies and other species-to-species differences. Furthermore, many fatality monitoring studies are not long enough to allow the full range of affected species to be detected [74], and this limitation affects the proportions of different species found at individual wind facilities. Additionally, FT may be overestimated because estimates of adult survival used in the denominator rarely included fatality from wind turbines because the studies were typically done in areas without turbines or prior to turbine installation. We believe the bias arising from this limitation is slight because fatality from wind turbines was usually vanishingly small compared to overall estimates of fatality.

When applying this prioritization approach, the best available data must be used. This will vary by study area and species. In our case, state wildlife action plans represent knowledge about the conservation status of many species, yet these are not updated annually. Furthermore, the relative abundance maps based on BBS use data from 2006 to 2010 and thus represent an average distribution across 5 years of variability. We felt these represented reasonably good estimates of current conservation status and distribution, but note that in some cases, it may be necessary to generate the input data required by the approach.

Possingham et al. (see page 506 in [75]) concluded that it is “naïve and counterproductive from all points of view to use threatened species lists alone to allocate resources for recovery, to guide reserve planning, or to constrain the use of the natural environment. Other tools are necessary for these tasks, and threatened species lists should be a part of the contributing information.” There are several limitations to the prioritization we developed and we acknowledge these issues. However, the problem of predicting population-level consequences from wind energy, particularly when considering a future dominated by renewable energy, is both extremely vexing and important. As such, we present the prioritization approach with the perspective that it is one of many iterations towards an improved assessment approach and urge others to improve upon it.

Our national-scale and multi-species approach to prioritization of population-level effects of wind energy development is the first to incorporate multiple components of risk and to consider robustness of priority rankings to the setting of priority category criteria. The outputs of our assessments should be useful for ecological researchers, conservation organizations, agencies, and the wind industry for targeting future research inquiry, conservation efforts, and efforts to mitigate population effects. Such rapid prioritization approaches are a crucial first step to conservation given the current speed and scale of wind energy expansion and the increased preponderance of other types of anthropogenic threats to wildlife.

## Supporting Information

S1 Table Habitat classifications. Aggregated habitat classifications from IUCN used to determine the number of suitable habitat types, *h*, for each species to calculate Indirect Risk Index.
(XLSX)

S2 Table Species data. Information used to assign priority categories for 428 avian species that occur in the conterminous United States.
(XLSX)
